# Supplementary material for: A large-scale in silico replication of ecological and evolutionary studies
Source: Nat Ecol Evol. 2024 Sep 26;8(12):2179–83. doi: 10.1038/s41559-024-02530-5 (PMC11618063; doi:10.1038/s41559-024-02530-5)
Supplement: Supplementary file 1 — Supplementary technical details of F-localization CIs with dependent replicates and reproducible R code. [file 41559_2024_2530_MOESM1_ESM.pdf]

nature ecology & evolution

Supplementary information

https://doi.org/10.1038/s41559-024-02530-5

A large-scale in silico replication of ecological and evolutionary studies

In the format provided by the authors and unedited

Supplementary technical details: F-Localization confidence intervals with dependent replicates

Overview

Below we follow the notation in Ignatiadis and Wager [2022a]. Therein, the authors propose confidence intervals for an empirical Bayes analysis wherein the statistician observes independent pairs (μ_i, Z_i), $i = 1, \ldots, n$, distributed as follows:

$$\mu_i \sim G, \;\; Z_i \mid \mu_i \sim p(\cdot \mid \mu_i). \tag{1}$$

Above, the μ_i are unknown parameters distributed according to an unknown prior distribution G that lies in a convex class of distributions $\mathcal{G}$ and $p(\cdot \mid \mu_i)$ is a known likelihood, e.g, $Z_i \mid \mu_i \sim \mathrm{N}(\mu_i, 1)$, or $\mu_i \sim |\mathrm{N}(\mu_i, 1)|$. Interest lies in forming confidence intervals for functionals $\theta(G)$ of the unknown distribution G, with an emphasis on linear and ratio-form functionals of G. For example, the conditional probability of replication can be expressed as a ratio-form functional.

Below, we provide an extension of the Dvoretzky–Kiefer–Wolfowitz (DKW) F-Localization confidence intervals described in Ignatiadis and Wager [2022a, Section 2] to the setting with dependent replicates. Our framework encompasses the following two settings.

Setting 1 (Single per-unit parameter, multiple dependent noise realizations)**.** *The i-th unit has parameter μ_i, $i = 1, \ldots, n$. We observe $K_i \in \mathbb{N}$ observations $Z_{i1}, \ldots, Z_{iK_i}$ from $p(\cdot \mid \mu_i)$:*

$$\mu_i \sim G, \;\; Z_{ij} \mid \mu_i \sim p(\cdot \mid \mu_i), \;\; j = 1, \ldots, K_i.$$

We assume independence across units i. However, the observations $Z_{ij}, j = 1, \ldots, K_i$, may be arbitrarily dependent conditional on μ_i.

Setting 2 (Cluster dependence)**.** *We use i ($i = 1, \ldots, n$) to index a cluster of K_i dependent units with parameters $\mu_{i1}, \ldots, \mu_{iK_i}$ and observations $Z_{i1}, \ldots, Z_{iK_i}$ such that:*

$$\mu_{ij} \sim G, \;\; Z_{ij} \mid \mu_{ij} \sim p(\cdot \mid \mu_{ij}), \;\; j = 1, \ldots, K_i.$$

We assume independence across clusters i. However, the pairs $(\mu_{ij}, Z_{ij}), j = 1, \ldots, K_i$, may be arbitrarily dependent (that is, both the parameters may be dependent, and the noise conditional on the parameters may be dependent).

Formally, Setting 1 is a special case of Setting 2, wherein we set $\mu_{i1} = \ldots = \mu_{iK_i} = \mu_i$. The possibility of extending some of the results in Ignatiadis and Wager [2022a] to Setting 2 (cluster dependence) was suggested in Ignatiadis and Wager [2022b] following a question raised by Xie and Stephens [2022].

Review of F-Localization approach

We first review the general F-Localization proposal of Ignatiadis and Wager [2022a]. Let

$$F_G(z) := \int p(z \mid \mu)\, dG(\mu) \tag{2}$$

be the marginal distribution of Z under the distribution G of μ. The F-Localization approach at confidence level $1-\alpha$ for $\alpha \in (0,1)$ proceeds in three steps:

1. Use the observed data to form a $(1-\alpha)$-confidence set of distributions $\mathcal{F}(\alpha)$ with the property that $\mathbb{P}_G\left[F_G \in \mathcal{F}(\alpha)\right] \geq 1-\alpha$ for all $G \in \mathcal{G}$. The set $\mathcal{F}(\alpha)$ is called an "F-Localization."

2. Use the F-Localization to constrain the plausible priors $G \in \mathcal{G}$. In particular, form a confidence set for $G \in \mathcal{G}$ by restricting attention to distributions G such that $F_G \in \mathcal{F}(\alpha)$, where F_G is defined in (2).

3. Finally, construct confidence intervals for the estimand $\theta(G)$ by computing the infimum and supremum of $\theta(G)$ over all $G \in \mathcal{G}$ such that $F_G \in \mathcal{F}(\alpha)$:

$$\hat{\theta}_- = \inf\left\{\theta(G)\,:\, G \in \mathcal{G},\ F_G \in \mathcal{F}(\alpha)\right\}, \quad \hat{\theta}_+ = \sup\left\{\theta(G)\,:\, G \in \mathcal{G},\ F_G \in \mathcal{F}(\alpha)\right\}. \tag{3}$$

   For common estimands, the above can be computed using convex optimization. For example, for the Dvoretsky-Kiefer-Wolfowitz (DKW) F-Localization, the infimum and supremum are computed by solving a linear programming problem (see Ignatiadis and Wager [2022a] for details).

If $\mathcal{F}(\alpha)$ is indeed an F-Localization, then it follows that $\mathcal{I} = [\hat{\theta}_-, \hat{\theta}_+]$ is a $(1-\alpha)$-confidence interval for $\theta(G)$, that is, $\mathbb{P}_G\left[\theta(G) \in \mathcal{I}\right] \geq 1-\alpha$ for all $G \in \mathcal{G}$.

F-Localization for dependent replicates

Below we propose an F-Localization for the case of dependent replicates as described in Settings 1 and 2. Once the F-Localization has been constructed, the computation for Steps 2 & 3 proceeds precisely as in Ignatiadis and Wager [2022a, Section 2].

Our goal is to propose an F-Localization that generalizes Ignatiadis and Wager [2022a, Equation 16]. To this end, first we define the empirical distribution function of the $(Z_{ij})_{ij}$, wherein we assign a weight to each observation Z_{ij} of the i-th unit/cluster that is inversely proportional to the number of observations K_i.

$$\widehat{F}(z) := \frac{1}{n}\sum_{i=1}^{n}\frac{1}{K_i}\sum_{j=1}^{K_i}\mathbb{1}(Z_{ij} \leq z), \quad z \in \mathbb{R}. \tag{4}$$

We then construct an F-Localization by forming a Kolmogorov-Smirnov band around $\widehat{F}$. The band is defined as follows:

$$\mathcal{F}(\alpha) := \left\{F \text{ distribution on } \mathbb{R}\,:\, \sup_{z\in\mathbb{R}}\left|\widehat{F}(z) - F(z)\right| \leq \sqrt{\log(2e/\alpha)/(2n)}\right\}, \tag{5}$$

The following proposition demonstrates that (5) is a valid F-Localization under both settings.

Proposition 1. *Under both Setting 1 and Setting 2, it holds that $\mathcal{F}(\alpha)$ defined in (5) is a valid F-Localization:*

$$\mathbb{P}_G\left[F_G \in \mathcal{F}\right] \geq 1 - \alpha.$$

Proof. First, let

$$\xi_i \overset{ind.}{\sim} \text{Unif}\left\{1, \ldots, K_i\right\},$$

and then define $Z_i' := Z_{i\xi_i}$ and $\mu_i' := \mu_i$ (in Setting 1) or $\mu_i' := \mu_{i\xi_i}$ (in Setting 2).

In this way, we have thrown out all but one observation from each unit/cluster. We find ourselves in the setting with a single replicate as in (1):

$$\mu_i' \sim G, \;\; Z_i' \mid \mu_i' \sim p(\cdot \mid \mu_i').$$

Next, consider the empirical distribution function of the Z_i':

$$\widehat{F}(z; \xi_1, \ldots, \xi_n) := \frac{1}{n}\sum_{i=1}^{n} \mathbb{1}(Z_i' \leq z).$$

Then, by Massart's tight constant for the Dvoretzky–Kiefer–Wolfowitz (DKW) inequality [Massart, 1990], we have that for every $\varepsilon > 0$:

$$\mathbb{P}_G\left[\sup_{z \in \mathbb{R}}\left|\widehat{F}(z; \xi_1, \ldots, \xi_n) - F_G(z)\right| > \varepsilon\right] \leq 2\exp(-2n\varepsilon^2). \tag{6}$$

Let us write $\mathbb{E}_\xi\left[\cdot\right]$ for the expectation with respect to only the auxiliary random variables $\xi_1, \ldots, \xi_n$ and observe that:[1]

$$\widehat{F}(\cdot) = \mathbb{E}_\xi\left[\widehat{F}(\cdot; \xi_1, \ldots, \xi_n)\right].$$

Now take any function Φ that is convex and increasing. We have that:

$$\begin{aligned}
&\mathbb{E}_G\left[\Phi\left(\sup_{z \in \mathbb{R}}\left|\widehat{F}(z) - F_G(z)\right|\right)\right] \\
&= \mathbb{E}_G\left[\Phi\left(\sup_{z \in \mathbb{R}}\left|\mathbb{E}_\xi\left[\widehat{F}(z; \xi_1, \ldots, \xi_n) - F_G(z)\right]\right|\right)\right] \\
&\leq \mathbb{E}_G\left[\Phi\left(\mathbb{E}_\xi\left[\sup_{z \in \mathbb{R}}\left|\widehat{F}(z; \xi_1, \ldots, \xi_n) - F_G(z)\right|\right]\right)\right] \\
&\leq \mathbb{E}_G\left[\Phi\left(\sup_{z \in \mathbb{R}}\left|\widehat{F}(z; \xi_1, \ldots, \xi_n) - F_G(z)\right|\right)\right].
\end{aligned}$$

For the penultimate inequality, we used the monotonicity of Φ, and for the last inequality, we used Jensen's inequality. Since Φ was an arbitrary increasing and convex function, it follows by Panchenko [2003] and (6) that for any $\varepsilon > 0$:

$$\mathbb{P}\left[\sup_{z \in \mathbb{R}}\left|\widehat{F}(z) - F_G(z)\right| > \varepsilon\right] \leq 2e\exp(-2n\varepsilon^2).$$

This implies the claim of the proposition. ∎

[1] In contrast, we use the notation $\mathbb{E}_G\left[\cdot\right]$ to refer to the expectation with respect to all sources of randomness including ξ_i, μ_{ij} (or μ_i) and Z_{ij}. The subscript G is used to indicate that the expectation depends on G

References

N. Ignatiadis and S. Wager. Confidence intervals for nonparametric empirical Bayes analysis (with discussion). *Journal of the American Statistical Association*, 117(539):1149–1166, 2022a.

N. Ignatiadis and S. Wager. Rejoinder: Confidence intervals for nonparametric empirical Bayes analysis. *Journal of the American Statistical Association*, 117(539):1192–1199, 2022b.

P. Massart. The tight constant in the Dvoretzky–Kiefer–Wolfowitz inequality. *The Annals of Probability*, pages 1269–1283, 1990.

D. Panchenko. Symmetrization approach to concentration inequalities for empirical processes. *The Annals of Probability*, 31(4):2068–2081, 2003.

D. Xie and M. Stephens. Discussion of "Confidence intervals for nonparametric empirical Bayes analysis". *Journal of the American Statistical Association*, 117(539):1186–1191, 2022.

Reproducible R code

Table of Contents

Packages 2
Data 3
Modelling 5
  z-statistic 5
  SNR 7
  Joint distribution 7
Estimates 9
  Power 9
  Replication 10
  Sample size multiplier 14

Packages

```r
#knitr::opts_chunk$set(fig.height = 8, fig.width = 10)
set.seed(2024)
suppressPackageStartupMessages({
  library(dplyr)
  library(readr)
  library(tidyr)
  library(ggplot2)
  library(aplot)
  library(ggplotify)
  library(here)
  library(patchwork)
  library(plot3D)
  })
# functions
source(here("func","func.R"))
```

Data

The database comprised 466 meta-analytic datasets and 88,218 observations of ecological and evolutionary effects, curated by Costello and Fox. These datasets were obtained through a systematic search of meta-analysis papers indexed in Web of Science categories relevant to ecology and evolutionary biology. We eliminated effect size estimates with zero and missing sampling variances.

The 466 meta-analytic datasets encompassed diverse research topics within ecology and evolutionary biology. However, it is important to realize that the trials in our dataset are not a random sample of all the trials in the research field, and may therefore not be entirely representative.

Furthermore, it is well known that trials that do not reach statistical significance have a lower chance of publication. We do not know the extent of this "publication bias" and we do not take it into account.

Import the main data (the `.csv` file named `main_dat.csv`).

```
dat_all <- read.csv(here("data/main","main_dat_processed.csv"))
head(dat_all)
```

```
##   paper.id      meta.analysis.paper meta.analysis.id meta.analysis.y
ear
## 1        1 bird.et.al.2019.ecoletts                1               2
019
## 2        1 bird.et.al.2019.ecoletts                1               2
019
## 3        1 bird.et.al.2019.ecoletts                1               2
019
## 4        1 bird.et.al.2019.ecoletts                1               2
019
## 5        1 bird.et.al.2019.ecoletts                1               2
019
## 6        1 bird.et.al.2019.ecoletts                1               2
019
##         study study.year eff.size.measure grouped_es   eff.size var.e
ff.size
## 1 Harrison_1       1964         hedges.d        SMD -1.7771359    0.
1099483
## 2 Harrison_1       1964         hedges.d        SMD -1.7519846    0.
2385344
## 3 Harrison_1       1964         hedges.d        SMD -1.3276771    1.
7153664
## 4 Harrison_1       1964         hedges.d        SMD -1.3276771    1.
7153664
## 5 Harrison_1       1964         hedges.d        SMD -0.3359459    0.
4703529
## 6 Harrison_1       1964         hedges.d        SMD -0.6486920    1.
0548881
```

```
##   se.eff.size          z              key        study2   dup
## 1   0.3315845 -5.3595265 1_Harrison_1_1964 harrison_1964 FALSE
## 2   0.4883999 -3.5871930 1_Harrison_1_1964 harrison_1964 FALSE
## 3   1.3097200 -1.0137106 1_Harrison_1_1964 harrison_1964 FALSE
## 4   1.3097200 -1.0137106 1_Harrison_1_1964 harrison_1964 FALSE
## 5   0.6858228 -0.4898436 1_Harrison_1_1964 harrison_1964 FALSE
## 6   1.0270775 -0.6315902 1_Harrison_1_1964 harrison_1964 FALSE

# make a copy
d <- dat_all
nrow(d)                              # number of estimates

## [1] 88218

length(unique(d$study2))             # number of unique studies

## [1] 12927

length(unique(d$meta.analysis.id))  # number of meta-analyses

## [1] 466
```

We plot the *z*-statistics distribution:

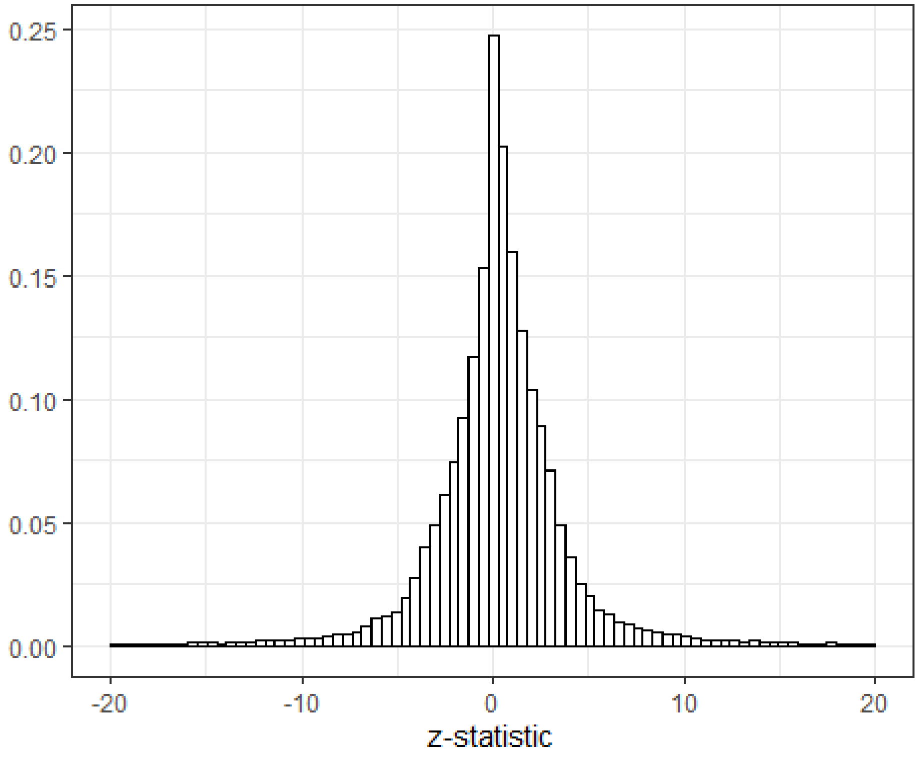

Supplementary Figure 1. The histogram of the observed z statistics representing test statistics of 88,218 ecological and evolutionary effects. The z statistic is equal to effect size estimate $\overline{ES}$ divided by its standard error SE ($\overline{ES}/SE$) under the standard normal distribution.

Modelling

For each observation in the database, let ES denote the (unobservable!) true or population effect size, and $\overline{ES}$ be the observed effect size. We assume that $\overline{ES}$ a normally distributed, unbiased estimate of the true effect ES with a known sampling standard deviation (aka standard error) SE. So,

$$\overline{ES} \sim {\cal N}(ES,SE^2)$$

The main effect size measures in our database are standardized mean differences (SMD; 45%), log-transformed response ratios (lnRR; 36%), and Fisher's z-transformed correlations (15%). For these measures, it is reasonable (and customary) to assume normality. The z-statistic is defined as

$$z = \overline{ES}/SE$$

If the absolute value of the z-statistic exceed 1.96 then the observed effect is "statistically significantly" different from zero at significance level $\alpha = 0.05$ (two-sided).

Finally, we define the signal-noise-ratio (SNR) as the strength of true effect size (the signal) relative standard error of the estimate (the noise),

$$SNR = ES/SE$$

It follows from our assumptions that

$$z \sim {\cal N}(SNR,1)$$

In other words, we can think if the z-statistic as the sum of the signal-to-noise ratio and a standard normal error term.

z-statistic

We start by estimating the distribution of the z-statistics across the trials in our dataset. We use the method of maximum likelihood. We will assume that the z-statistics follow a mixture of zero-mean normal distributions. We can write this distribution as

$$g(z) = \sum_{i=1}^{4} p_i \, \varphi(z/\sigma_i)/\sigma_i$$

where φ is the density of the standard normal distribution, and the mixture probabilities p_i (or "weights") are non-negative and sum to one. That is, p_i and $\sum_{i=1}^{4} p_i = 1$.

By fitting a symmetric distribution to the observed z-statistics, we are ignoring their signs. Put differently, by fitting a mixture of zero-mean normals to the z-stats, we are essentially fitting a mixture of half-normals to the *absolute* z-stats.

Since the z-statistics are the sum of the signal-to-noise ratio and a standard normal error term, the standard deviations σ_i of the mixture components must be at least 1. So, we run a constrained optimization to find the maximum likelihood estimates. The constraints are:

- $p_i \geq 0, \quad (i = 1,2,3,4)$
- $\sum_{i=1}^{4} p_i = 1$
- $\sigma_i \geq 1, \quad (i = 1,2,3,4)$

Some studies have multiple outcomes, so we weight each study inversely proportional to how often it appears in our data set.

```
# count number of outcomes per study
d = group_by(d,study2) %>% mutate(count=n(),w=1/count)
# censor two extreme z values at, say, -100 and 100, respectively
d$z[d$z == max(d$z)] = 100
d$z[d$z == min(d$z)] = -100

#df=mix(z=d$z,k=4,c1=0,c2=10,weights=d$w)
#write.csv(df, here("data/model","df_main.csv"), row.names = F)
df=read.csv(here("data/model","df_main.csv")) # to save time, we upload
 the pre-fitted model
p=df$p
m=df$m
sigma=df$sigma
round(df,2)

##      p m sigma
## 1 0.21 0  2.53
## 2 0.17 0  1.00
## 3 0.43 0  2.56
## 4 0.19 0  8.47
```

We plot the *weighted* histogram of the absolute z-statistics together with the fitted mixture:

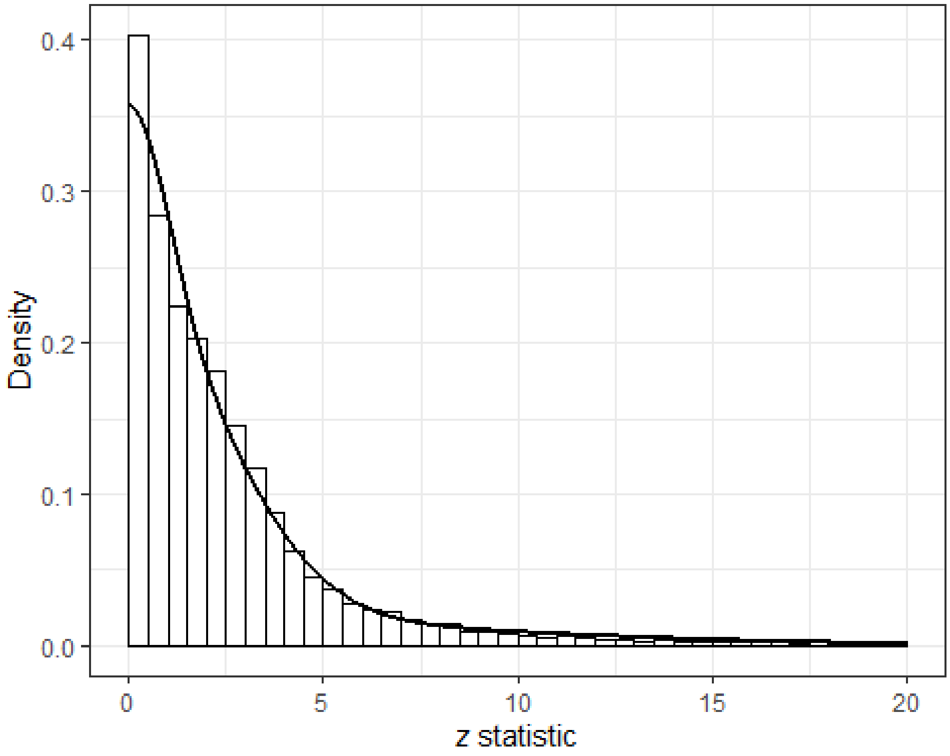

Supplementary Figure 2. The histogram of 88,218 z statistics with the density derived from the fitted mixture of 4 zero-mean normal distributions.

We see that the mixture fits quite well.

SNR

As we discussed, the z-statistics are the sum of the signal-to-noise ratio and a standard normal error term. Therefore it is easy to obtain the distribution of the SNRs from the distribution of the *z*-statistics. We simply subtract 1 from variances of the mixture components. So, the distribution of the SNRs can be written as

$$f(x) = \sum_{i=1}^{4} p_i \, \varphi(x/\tau_i)/\tau_i$$

where $\tau_i = \sqrt{\sigma_i^2 - 1}$.

Joint distribution

We have now estimated the marginal distributions of the z-statistics and the SNRs. But we also know the *conditional* distribution of the z-statistic given the SNR (it's normal with mean SNR and standard deviation 1). This means that we have the *joint* distribution of the z-statistics the SNRs.

Finally, we can use the well-known theory of bivariate normal distributions to obtain the conditional distribution of the SNR given the observed z-statistic.

Estimates

We can use the estimated joint distribution of the z-statistics and the SNRs to calculate the statistical power and replicability for the 111,321 observed ecological and evolutionary effects.

Power

The "achieved" or "actual" power is simply the probability of reaching statistical significance (two-sided, level $\alpha = 0.05$). Obviously, this probability depends on the true effect size and on the standard error of the estimate. Under our assumptions, this can be expressed in terms of the SNR.

$$\text{power}(SNR) = \Phi(-1.96 - SNR) + 1 - \Phi(1.96 - SNR).$$

where Φ is the cumulative distribution function (CDF) of the standard normal distribution. Once again: This is the power against the *true* effect. Of course, the true effect is never known. However, since we can estimate the distribution of the SNR across the studies in our dataset, we can also estimate the distribution of the power across a large number of studies.

Note 1: The power against the true effect should not to be confused with the power the study is designed for. Most studies are designed to have 80% or 90% against some effect that is considered important or plausible (or both).

Note 2: The power against the true effect should also not be confused with the "observed" of "post hoc" power.

Since the power against the true effect is a function of the SNR, we can obtain its distribution by transforming the distribution of the SNR. We generate a sample of size 10^7 from the (estimated) distribution of the SNRs, and then we apply the transformation to get a sample from the distribution of the power.

```
snr=rmix(10^7,p=p,m=m,s=sqrt(sigma^2 - 1))
power=pnorm(-1.96,snr,1) + 1 - pnorm(1.96,snr,1)
df=data.frame(power=power)
```

We can now show the power distribution with a histogram:

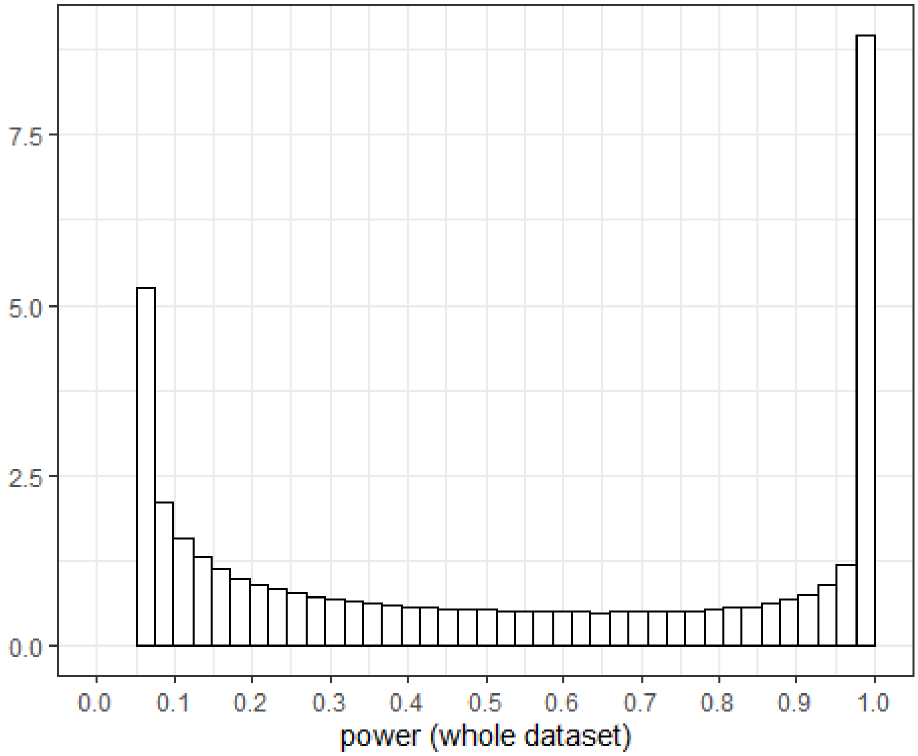

Supplementary Figure 3. Histogram of a sample of size 10^7 from the estimated distribution of the power against the true effect.

Based on the estimated distribution of the SNR, the average power can be calculated.

```
summary(power)

##    Min. 1st Qu.  Median    Mean 3rd Qu.    Max.
##  0.0500  0.0619  0.3128  0.4440  0.8780  1.0000
```

We also have a direct estimate of the mean power, namely the weighted proportion of significant results.

```
sum(d$w*(abs(d$z)>1.96))/sum(d$w)

## [1] 0.4586247
```

Replication

Suppose we have conducted a study and obtained the z-statistic z. Now consider a (hypothetical) replication study with exactly the same design (the same effect, sample size, standard error, etc.) Now consider the event that the replication is "successful" in the sense that it reaches statistical significance in the same direction as the original study, i.e.

$$z \times z_{\text{repl}} > 0 \text{ and } |z_{\text{repl}}| > 1.96$$

Using simulation, we can easily compute mean replication for the whole data set, both unconditionally and conditionally on statistical significance

```
snr=rmix(10^7,p=p,m=m,s=sqrt(sigma^2 - 1))
z.orig=snr + rnorm(10^7) # original
z.repl = snr + rnorm(10^7) # replication
replicate=(z.orig * z.repl > 0) & (abs(z.repl) > 1.96)
mean(replicate)                      # unconditional probability of repli
cation

## [1] 0.428384

mean(replicate[abs(z.orig)>1.96])  # conditional probability of replica
tion given |z|>1.96

## [1] 0.7692444
```

We can also compute the conditional probability of "successful replication" given the absolute value of the z-statistic of the original study. The symmetry of the distribution of the z-statistic implies that the conditional probability of "successful replication" given $|Z| = z$ is equal to the conditional probability given $Z = z$.

```
replicate=sapply(d$z,replcalc,p=p,m=m,s=sqrt(sigma^2-1))
```

We can verify the unconditional and conditional probability of "successful replication" by averaging over the empirical distribution of the observed z-statistics.

```
sum(d$w*replicate)/sum(d$w)   # unconditional probability of replicatio
n

## [1] 0.4297694

ind=which(abs(d$z)>1.96)
sum(d$w[ind]*replicate[ind])/sum(d$w[ind])  # conditional probability o
f replication given |z|>1.96

## [1] 0.7592826
```

Replication rate profile:

```
# pdf of the z-stats
density=dmix(d$z,p,m,sigma)

df=data.frame(z=abs(d$z),replicate=replicate,density=density)

replicate.dist <- ggplot(data=df, aes(x = z, y = replicate)) +
  geom_line(linetype="dashed") +
  geom_line(data=filter(df,z>1.96),linetype="solid") +
  scale_y_continuous(limits = c(0, 1), breaks = seq(0, 1, by = 0.2),
                     minor_breaks=seq(0,1,0.05),
```

```
                     expand = expansion(mult = c(0, 0.01))) +
  scale_x_continuous(limits = c(0, 10), breaks = seq(0, 10, by = 2),
                     expand = expansion(mult = c(0, 0.01))) +
  ylab("Replication rate") + xlab(expression(paste(italic(z)~"statistic
"))) +
  labs(colour = NULL) +
  theme_bw() +
  theme(axis.title = element_text(size = 14),
        axis.text = element_text(size = 12))

top.dist2 <- ggplot(data=df, aes(x = z,y=density)) +
  geom_line(linetype="dashed")+
  geom_ribbon(data=filter(df,z>1.96),aes(z, ymax=density, ymin=0),
              fill="grey") +
  geom_line(data=filter(df,z>1.96),linetype="solid") +
  scale_x_continuous(limits = c(0, 10), expand = expansion(mult = c(0,
0.01))) +
  labs(x = "", y = "") +
  theme_void() +
  theme(plot.margin = grid::unit(c(0, 0, 0, 0), "lines"))

replicate.top.dist <- suppressWarnings(as.ggplot(insert_top(
   replicate.dist, top.dist2, height = 0.5)))
replicate.top.dist + plot_layout(tag_level = 'new') +
  plot_annotation(tag_levels = list(c(''))) &
  theme(plot.tag = element_text(size = 16, face = "bold"))
```

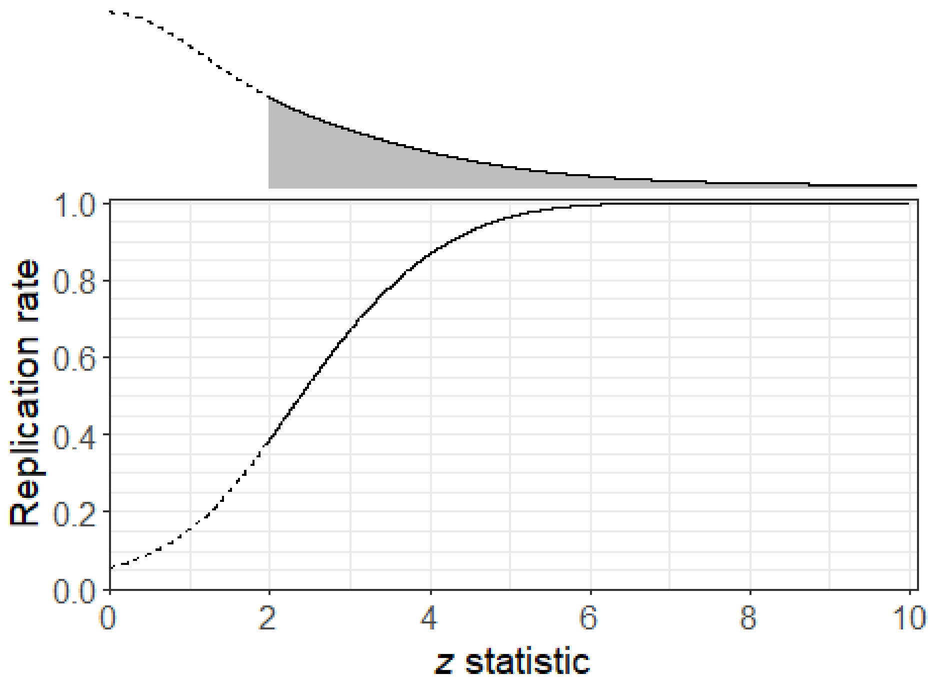

Supplementary Figure 4. The relationship between the probability of successful replication and the observed z statistic of the original study.

We show the conditional replication rates at selected z-values, re-formulated as the scales of evidence strength according to according to Bland M. 2015. An Introduction to Medical Statistics. Oxford, UK: Oxford Univ. Press. 4th ed. We covert z-values to two-sided p-values. Our purpose is to build up the intuitive relationship between the replication rates and evidence strength, albeit interpreting z-values (or, equivalently, p-values) as evidence strength is controversial (see **Notes**).

Notes:

The "scales of evidence" (like "no evidence", "weak evidence",…) are quite problematic, see Efron, B., Gous, A., Kass, R. E., Datta, G. S., & Lahiri, P. (2001). Scales of evidence for model selection: Fisher versus Jeffreys. Lecture Notes-Monograph Series, 208-256. It's even doubtful if p-values can be taken as measures of evidence at all. As Wasserstein puts it: "By itself, a p-value does not provide a good measure of evidence regarding a model or hypothesis." See Wasserstein, R. L., & Lazar, N. A. (2016). The ASA statement on p-values: context, process, and purpose. The American Statistician, 70(2), 129-133.

```
pval=c(0.1,0.05,0.01,0.001,0.0001)
strength = c("No evidence", "Weak evidence","Moderate evidence",
             "Strong evidence","Very strong evidence")
strength = factor(strength,
                  levels=c("No evidence", "Weak evidence",
                           "Moderate evidence","Strong evidence",
                           "Very strong evidence"))
zval=qnorm(1 - pval/2)
replicate=sapply(zval,replcalc,p=p,m=m,s=sqrt(sigma^2-1))
replicate=round(replicate,2)
replicate_typical <- data.frame(strength, replicate)
data.frame(strength,pval,zval,replicate)

##                 strength  pval     zval replicate
## 1          No evidence 1e-01 1.644854      0.29
## 2        Weak evidence 5e-02 1.959964      0.38
## 3    Moderate evidence 1e-02 2.575829      0.56
## 4      Strong evidence 1e-03 3.290527      0.74
## 5 Very strong evidence 1e-04 3.890592      0.85

# figure
replicate_typical.p <- ggplot(replicate_typical, aes(x = replicate, y =
 strength)) +
  geom_segment(aes(x = 0, y = strength, xend = replicate, yend = streng
th)) +
  geom_point(aes(size = replicate)) +
  scale_size(range=c(8,15)) +
```

```
  geom_text(aes(label = scales::percent(replicate)), size = 3.5, color
= "white") +
  guides(size = "none") +
  xlab("Replication rate") +
  labs(colour = NULL, y = "") +
  theme_bw() +
  theme(axis.title = element_text(size = 12),
        axis.text.x = element_text(size = 12),
        axis.text.y = element_text(size = 12),
        plot.margin=unit(c(0,0,0,0), 'cm')) +
  scale_x_continuous(limits = c(0, 1), breaks = seq(0, 1, by = 0.2),
                     minor_breaks=seq(0,1,0.05))

replicate_typical.p
```

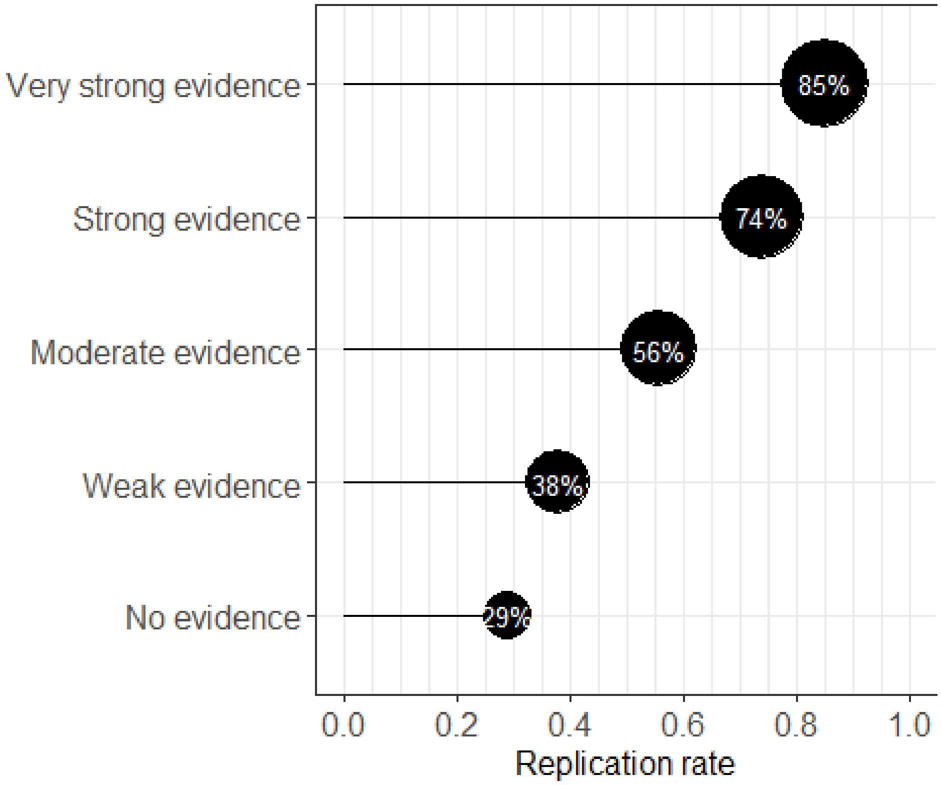

Supplementary Figure 5. The quantitative relationship between the replication probability and tentative evidence strength benchmarks.

Sample size multiplier

If the sample size of the replication study is twice as large as that of the original study, then the SNR of the replication study will be larger by a factor square root of 2. Thus, we can also compute the conditional probability of a successful replication

given the z-statistic of the original study when the replication study is larger (or smaller) by some factor.

Conditional replication rates when the original study has $z = 2$ or $z = 3.3$ and the replication study is $1,2, \ldots, 10$ times larger.

```
# calculate replication probabilities when multiplying the
# sample size of the original study with factors 1,2,...10.
replicate_rep_weak = sapply(1:10, function(x) replcalc(2, p=p, m=m, s=s
qrt(sigma^2-1), multiplier = x))
replicate_rep_strong = sapply(1:10, function(x) replcalc(3.3, p=p, m=m,
 s=sqrt(sigma^2-1), multiplier = x))

# prepare a data frame
replicate_multiplier <- data.frame(evidence = c(rep("Weak evidence", 10
),
                                                rep("Strong evidence",
10)),
                                   mult_values = rep(1:10, 2),
                                   replicate = c(replicate_rep_weak,
                                                 replicate_rep_strong))
# round
replicate_multiplier$replicate <- round(replicate_multiplier$replicate,
 2)
# data frame
replicate_multiplier

##           evidence mult_values replicate
## 1    Weak evidence           1      0.39
## 2    Weak evidence           2      0.55
## 3    Weak evidence           3      0.63
## 4    Weak evidence           4      0.68
## 5    Weak evidence           5      0.71
## 6    Weak evidence           6      0.74
## 7    Weak evidence           7      0.75
## 8    Weak evidence           8      0.76
## 9    Weak evidence           9      0.77
## 10   Weak evidence          10      0.78
## 11 Strong evidence           1      0.74
## 12 Strong evidence           2      0.89
## 13 Strong evidence           3      0.94
## 14 Strong evidence           4      0.96
## 15 Strong evidence           5      0.97
## 16 Strong evidence           6      0.97
## 17 Strong evidence           7      0.98
## 18 Strong evidence           8      0.98
## 19 Strong evidence           9      0.98
## 20 Strong evidence          10      0.98
```

```
# figure
replicate_multiplier.p <- replicate_multiplier %>%
  ggplot(aes(x = mult_values, y = replicate, color = evidence, fill = e
vidence)) +
  geom_point(color = "transparent") +
  geom_line(alpha=1, linewidth = 0.5) +
  scale_y_continuous(limits = c(0, 1), breaks = seq(0, 1, by = 0.2),
                     minor_breaks=seq(0,1,0.05),
                     expand = expansion(mult = c(0, 0.01))) +
  scale_x_continuous(limits = c(1, 10), breaks = seq(1, 10, by = 1),
                     labels=paste(1:10,"x",sep=''),
                     expand = expansion(mult = c(0.03, 0.03))) +
  ylab("Replication probability") +
  xlab(expression(paste("Relative sample size for the replication study
"))) +
  geom_point(data = replicate_multiplier, aes(size = replicate)) +
  #scale_size(range=c(8,15)) +
  geom_text(data = replicate_multiplier,
            aes(label = scales::percent(replicate)), size = 3.5, color
= "gray10") +
  guides(size = "none", color = "none", fill = "none") +
  labs(colour = NULL) +
  theme_bw() +
  theme(axis.title = element_text(size = 12),
        axis.text = element_text(size = 12),
        plot.margin=unit(c(0.2,0.3,0,0), 'cm')) +
  annotate("text", x = 3, y = 0.5, label = "Weak evidence", size = 3) +
  annotate("text", x = 3, y = 0.85, label = "Strong evidence", size = 3
)
replicate_multiplier.p
```

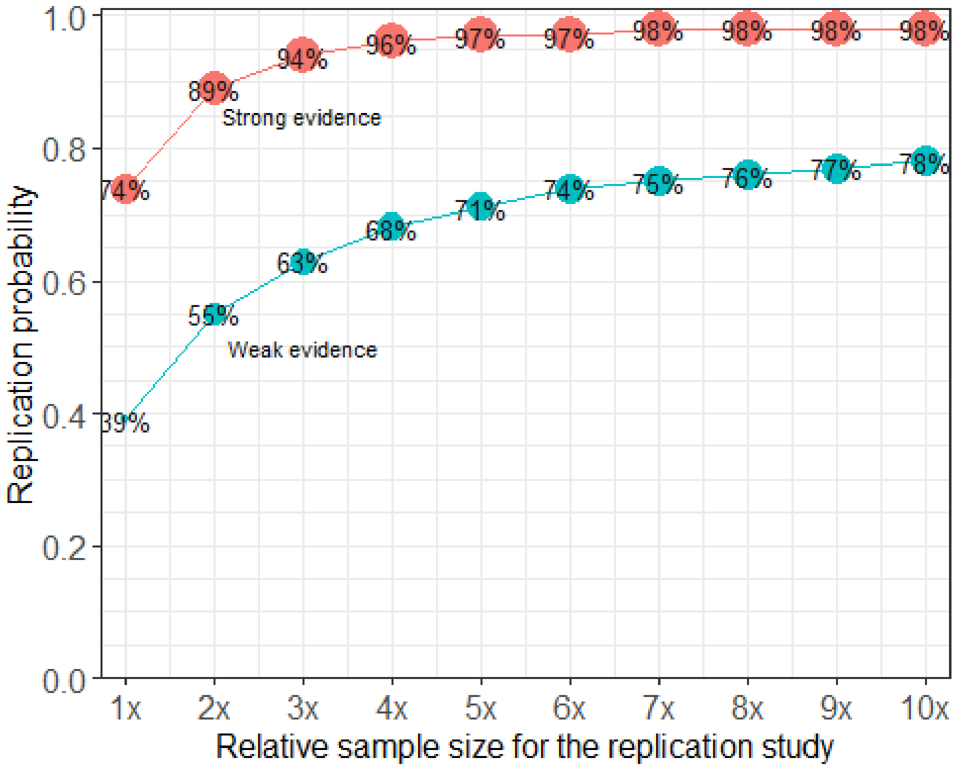

Supplementary Figure 6. The quantitative relationship between the replication probability and the relative sample size of the replication study compared to the sample size of the original study.
