# Supplementary material for: A large-scale in silico replication of ecological and evolutionary studies
Source: Nat Ecol Evol. 2024 Sep 26;8(12):2179–83. doi: 10.1038/s41559-024-02530-5 (PMC11618063; doi:10.1038/s41559-024-02530-5)
Supplement: Supplementary file 2 — Reporting Summary [file 41559_2024_2530_MOESM2_ESM.pdf]

nature portfolio

Corresponding author(s): Yefeng Yang

Last updated by author(s): Jul 22, 2024

Reporting Summary

Nature Portfolio wishes to improve the reproducibility of the work that we publish. This form provides structure for consistency and transparency in reporting. For further information on Nature Portfolio policies, see our Editorial Policies and the Editorial Policy Checklist.

Statistics

For all statistical analyses, confirm that the following items are present in the figure legend, table legend, main text, or Methods section.

n/a	Confirmed	
☐	☒	The exact sample size (*n*) for each experimental group/condition, given as a discrete number and unit of measurement
☒	☐	A statement on whether measurements were taken from distinct samples or whether the same sample was measured repeatedly
☐	☒	The statistical test(s) used AND whether they are one- or two-sided<br>*Only common tests should be described solely by name; describe more complex techniques in the Methods section.*
☐	☒	A description of all covariates tested
☐	☒	A description of any assumptions or corrections, such as tests of normality and adjustment for multiple comparisons
☐	☒	A full description of the statistical parameters including central tendency (e.g. means) or other basic estimates (e.g. regression coefficient) AND variation (e.g. standard deviation) or associated estimates of uncertainty (e.g. confidence intervals)
☐	☒	For null hypothesis testing, the test statistic (e.g. *F*, *t*, *r*) with confidence intervals, effect sizes, degrees of freedom and *P* value noted<br>*Give P values as exact values whenever suitable.*
☒	☐	For Bayesian analysis, information on the choice of priors and Markov chain Monte Carlo settings
☒	☐	For hierarchical and complex designs, identification of the appropriate level for tests and full reporting of outcomes
☐	☒	Estimates of effect sizes (e.g. Cohen's *d*, Pearson's *r*), indicating how they were calculated

Our web collection on statistics for biologists contains articles on many of the points above.

Software and code

Policy information about availability of computer code

Data collection	All data are extracted from meta-analysis articles indexed in the WoS for the search term “meta-analys*” AND “ecol*” while limiting to categories potentially related to ecology (e.g., Ecology, Evolutionary Biology, Multidisciplinary Sciences). No software was used for data collection.
Data analysis	All data processes and analyses are done using R programming language (4.0.1) and Julia programming Language (1.10.0). The code for reproducing the results of this study are available at the GitHub repository (https://github.com/Yefeng0920/replication_EcoEvo_git). The code also can be found at Zenodo repository 12 (https://doi.org/10.5281/zenodo.12748092). We provide both the R code and Julia code. The reproducible R code in an interactive format (code chunks paired with results) also can be found at the website https://yefeng0920.github.io/replication_EcoEvo_git/).

For manuscripts utilizing custom algorithms or software that are central to the research but not yet described in published literature, software must be made available to editors and reviewers. We strongly encourage code deposition in a community repository (e.g. GitHub). See the Nature Portfolio guidelines for submitting code & software for further information.

Data

Policy information about availability of data

All manuscripts must include a data availability statement. This statement should provide the following information, where applicable:

- Accession codes, unique identifiers, or web links for publicly available datasets
- A description of any restrictions on data availability
- For clinical datasets or third party data, please ensure that the statement adheres to our policy

The data to reproduce the results of this study are available at the GitHub repository (https://github.com/Yefeng0920/replication_EcoEvo_git). The data also can be found at Zenodo repository (https://doi.org/10.5281/zenodo.12748092).

Research involving human participants, their data, or biological material

Policy information about studies with human participants or human data. See also policy information about sex, gender (identity/presentation), and sexual orientation and race, ethnicity and racism.

Reporting on sex and gender	NA
Reporting on race, ethnicity, or other socially relevant groupings	NA
Population characteristics	NA
Recruitment	NA
Ethics oversight	NA

Note that full information on the approval of the study protocol must also be provided in the manuscript.

Field-specific reporting

Please select the one below that is the best fit for your research. If you are not sure, read the appropriate sections before making your selection.

☐ Life sciences ☐ Behavioural & social sciences ☒ Ecological, evolutionary & environmental sciences

For a reference copy of the document with all sections, see nature.com/documents/nr-reporting-summary-flat.pdf

Ecological, evolutionary & environmental sciences study design

All studies must disclose on these points even when the disclosure is negative.

Study description	We summarize our data in terms of the “true” effect ES, the effect size estimate $\widehat{(ES)}$, and its standard error SE. While being careful to take the statistical dependence of multiple observations within the same study into account, we obtain the marginal distribution of the z statistics $z=\widehat{(ES)}/SE$ using a Gaussian mixture model. Next, we employ a statistical technique called “deconvolution” to estimate the marginal density of the signal-noise ratio $SNR=ES/SE$. As with earlier work, replicability here is defined as finding a statistically significant effect size in the same direction in an exact replication study (in silico replication). Since the true effects are unobservable, being able to estimate replicability is rather remarkable.
Research sample	All data are extracted from meta-analysis articles indexed in the WoS for the search term “meta-analys*” AND “ecol*” while limiting to categories potentially related to ecology (e.g., Ecology, Evolutionary Biology, Multidisciplinary Sciences).
Sampling strategy	The coverage of this dataset is comprehensive, obtained previously through a systematic search of meta-analyses indexed in Web of Science categories relevant to ecology and evolution, encompassing 88,218 effects from 12,927 primary studies across a diverse array of research topics within ecology and evolution.
Data collection	The database comprised 466 meta-analytic datasets of ecological and evolutionary effects, curated by The database comprised 466 meta-analytic datasets and 111,327 observations of ecological and evolutionary effects, curated by Costello and Fox (ref 10).
Timing and spatial scale	Meta-analysis papers published before 2022.
Data exclusions	full text, supplements, and appendices were reviewed to confirm that the paper addresses an ecological topic and determine if data on effect sizes and their sampling variances were available.
Reproducibility	The authors affirms that the manuscript is an honest, accurate, and transparent account of the study being reported, and no important aspects of the study have been omitted. The data and code to reproduce the results of this study are available at the GitHub repository (https://github.com/Yefeng0920/replication_EcoEvo_git). We provide both the R code and Julia code. The

reproducible R code in an interactive format (HTML) also can be found at the Supplementary material and https://yefeng0920.github.io/replication_EcoEvo_git/). The code also can be found at Zenodo repository (https://doi.org/10.5281/zenodo.12748092).

Randomization	NA
Blinding	NA

Did the study involve field work? ☐ Yes ☒ No

Reporting for specific materials, systems and methods

We require information from authors about some types of materials, experimental systems and methods used in many studies. Here, indicate whether each material, system or method listed is relevant to your study. If you are not sure if a list item applies to your research, read the appropriate section before selecting a response.

Materials & experimental systems

n/a	Involved in the study
☒	☐ Antibodies
☒	☐ Eukaryotic cell lines
☒	☐ Palaeontology and archaeology
☒	☐ Animals and other organisms
☒	☐ Clinical data
☒	☐ Dual use research of concern
☒	☐ Plants

Methods

n/a	Involved in the study
☒	☐ ChIP-seq
☒	☐ Flow cytometry
☒	☐ MRI-based neuroimaging

Plants

Seed stocks	NA
Novel plant genotypes	NA
Authentication	NA
